# Ultrasound-guided Lumbar Erector Spinae Plane Block For Postoperative Analgesia in Femur Fracture: A Pediatric Case Report

**DOI:** 10.7759/cureus.5148

**Published:** 2019-07-16

**Authors:** Onur Balaban, Rabia Koçulu, Tayfun Aydın

**Affiliations:** 1 Anesthesiology, Kutahya Health Sciences University, Kutahya, TUR

**Keywords:** lumbar ultrasonography, erector spinae plane block, femur fixation, postoperative analgesia

## Abstract

The erector spinae plane (ESP) block is a recently defined regional anesthesia technique which is considered as an effective method in postoperative multimodal analgesia. ESP block is usually performed at the thoracic region in pediatric patients, but it is also possible to perform ESP block at the lumbar region. Femur fracture is one of the most common procedures especially in pediatric orthopedic surgery where postoperative pain management is essential. We aim to present a case of effective postoperative analgesia provided by ultrasound-guided lumbar ESP block in a 6-year-old patient after femur fixation surgery.

## Introduction

Regional anesthesia techniques have been widely used in pediatric surgery to prevent postoperative pain. Ultrasound-guidance has provided a safe way for single injection and continuous techniques in regional anesthesia. In addition to rapid and painless recovery, when combined with general anesthesia, the use of intraoperative anesthetic and postoperative analgesic drugs can be reduced [1-2].

Erector spinae plane (ESP) block is a new regional anesthesia technique, which has been defined recently. This block provides analgesia by targeting the dorsal and ventral branches of the spinal nerves [3]. ESP blocks applied in the lumbar region have been previously reported for postoperative analgesia of lower extremity surgeries in adults [4-6]. In our case report, we present the implementation of ESP block in a pediatric patient, which provided excellent postoperative analgesia after femur fracture surgery.

## Case presentation

**Figure 1:**
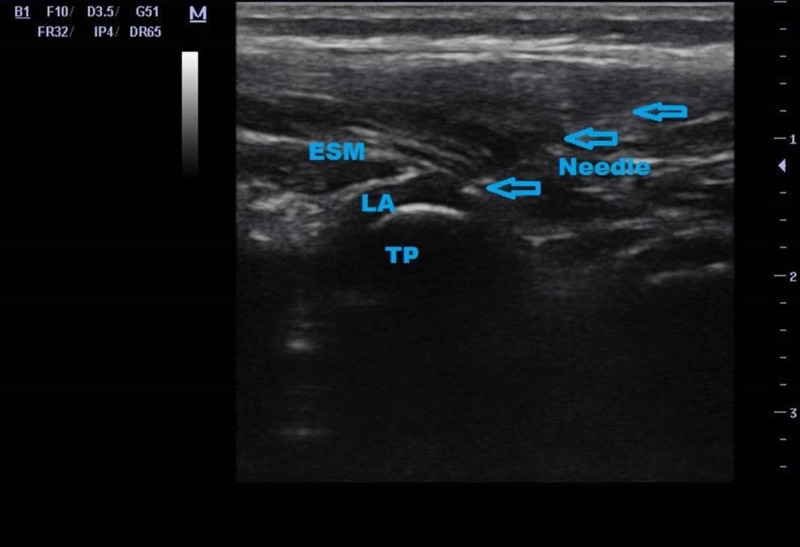
Ultrasound image of erector spinae plane block; blue arrows indicate needle ESM: erector spinae muscle; TP: transverse process; LA: local anesthetic.

A 6-year-old male (25 kg, 120 cm) was admitted to the emergency room with pain and restriction of movement in the right thigh after falling off the swing. After physical examination and X-ray imaging, a right femur shaft (medial zone) fracture was diagnosed. A fixation operation was planned and the patient was hospitalized in the Orthopedics and Traumatology Department. He had no co-morbid systemic disease, no history of allergy or anesthesia, but he had congenital one-kidney. The patient was assessed as ASA I according to American Society of Anesthesiologists (ASA) classification. General anesthesia was planned for the operation and a lumbar ESP block was planned for postoperative analgesia. Routine monitoring was applied to the patient including 3-channel electrocardiogram, noninvasive blood pressure, and pulse oximetry in the operation room; 20-gauge intravenous (IV) cannula was placed and dextrose 5% - NaCl 0.9% infusion (starting with 10 ml/kg, maintenance 5 ml/kg) was started. Midazolam 1 mg IV was administered for sedation and then routine general anesthesia protocol was applied. The internal fixation of femur lasted for two hours. A lateral incision was performed by the surgeons involving the dermatome levels L5. At the end of the operation, the patient was positioned in the left lateral for the ESP block. As the incision involved the dermatome level of L5, we preferred to make injection at the L5 vertebra level. After sterilization of the block area, a high-frequency linear probe (Mindray® Medical Electronics Co., Ltd. Shenzhen, China) was placed in paramedian sagittal orientation, 2 cm lateral to the midline at the L5 vertebrae level. The erector spinae muscle and the transverse process of the L5 vertebrae were visualized. A 22-gauge/5-cm block needle (Stimuplex®, Braun AG, Melsungen, Germany) was advanced cranial to caudal direction using in-plane technique and the local anesthetic solution was injected between the erector spinae muscle and the transverse process. A total of 20 ml bupivacaine 0.25% was used. Cranio caudal spread of the local anesthetic solution was visualized by ultrasound (Figure 1).

Acetaminophen 10 mg/kg was administered intravenously before the end of the operation. There were no complications during the procedure. Thereafter, the patient was extubated and admitted to the recovery room.

In the postoperative course, the patient's pain was evaluated according to the Wong-Baker Face Scale. In 30 minutes postoperatively, the patient had a pain intensity score 0 and was transferred to the ward after her vital signs and hemodynamic values were normal. The patient needed analgesics (250 mg acetaminophen IV) only once at the 12th postoperative hour (POH) during the follow-up period. Approximately 28 hours after the operation, the patient was mobilized and there was no increase of pain. There was also no requirement of opioids during the postoperative course. Wong-Baker Face score of the patient was 0 until the 12th POH. The pain score was evaluated as 2 after the 12th POH until the 24th POH.

## Discussion

Pediatric postoperative pain management may be challenging and requires a multimodal approach. Regional anesthesia techniques in the pediatric population are being used frequently in postoperative pain management due to the increased experience and accessibility of ultrasonography. When used in combination with general anesthesia, regional analgesia techniques reduce intra-operative and postoperative opioids and provide good postoperative pain control [1,2,7-8]. In our case, we managed to minimize postoperative pain by performing a single injection ESP block in combination with IV acetaminophen for postoperative analgesia in femur fracture surgery.

ESP block is a new regional anesthesia technique originally defined by Forero et al. for chronic thoracic pain management. This application provides the blockage of visceral and somatic nerve fibers by injecting the local anesthetic solution between the erector spinae muscle and the transverse process of the vertebrae [3]. Studies on cadavers have shown that injected local anesthesia for ESP block affects multiple dermatomes by spreading extensive cranio caudal vertebral levels. This is advantageous because it provides a wide blockage by single injection [9-10]. Lumbar ESP block was first used by Tulgar et al. for postoperative pain in an 86-year-old patient who underwent total hip arthroplasty at the L4 level [4]. Then ESP block was used in the literature for postoperative analgesia at L4 level for hip surgery, femoral surgery, and knee surgery [5-6]. Alici et al. performed single injection lumbar ESP block using a high volume for chronic pain in a patient with herpes zoster at lower extremity [11]. We have observed sufficient analgesia with a single injection of 20 ml of local anesthetic solution at the L5 vertebra level in our case. The intervention site is far away from structures such as major vascular vessels and nerve roots as so allowing the process to be performed safely.

According to a review by Tsui et al., there are many case reports in the literature while the clinical studies about ESP block are insufficient. Also, it was reported that ESP block was used in 90.9% of cases for multimodal analgesia [12].

Data regarding the use of ESP blocks in pediatric patients are limited and the use at thoracic levels has been reported previously. Munoz et al. presented ESP block in a 7-year-old patient with oncological thoracic surgery and reported successful postoperative analgesia [13]. Another case was an 11-year-old patient with an opioid allergy who was planned a laparoscopic cholecystectomy. In this case, a non-opioid analgesia technique was required and the peri-operative and postoperative analgesia were managed by using ESP block [14]. It has been shown to provide effective postoperative analgesia in incisional hernia repair and nephrectomy operations [15-16]. The ESP block for the lumbar region was performed at L2 level in a 4-year-old patient undergoing hip surgery by Elkoundi et al. [17]. This application has been shown to reduce the patient's analgesic need. Our aim was to provide effective postoperative analgesia in femur surgery, which is a very painful procedure in the postoperative period. Therefore, we performed the ESP block at the end of the operation, under general anesthesia. In the literature, relatively high doses were used for extensive spread of local anesthetic in order to ensure wide and sufficient analgesic effect [11,18]. We used 20 ml of bupivacaine 0.25% (0.8 ml/kg) which provided effective and prolonged postoperative analgesia. In our case, there was no need for opioids postoperatively (no requirement was asked by the patient) by using single injection ESP block applied at L5 level.

## Conclusions

When added to multimodal analgesia, single-injection lumbar ESP block provided effective postoperative analgesia after femur fracture surgery in our case. ESP blocks may be a feasible regional anesthesia method in the pediatric age group for analgesia in lower extremity surgery when performed at the lumbar vertebra level. Prospective controlled clinical studies should be performed to test its superiority to other regional anesthesia techniques.
